# From Assessing Workplace Spirituality to Psychological Safety: A Conceptual Framework for Healthcare Professionals’ Well-Being

**DOI:** 10.3390/healthcare14203552

**Published:** 2026-10-21

**Authors:** Panagiota Tragantzopoulou, Vaitsa Giannouli

**Affiliations:** 1 School of Social Sciences, University of Westminster, 115 New Cavendish St., London W1W 6UW, UK; g.tragantzopoulou@westminster.ac.uk; 2 Department of Psychology, Democritus University of Thrace, 691 00 Didymoteicho, Greece

**Keywords:** workplace spirituality, spiritual leadership, psychological safety, psychosocial safety climate, healthcare professionals, workforce sustainability, burnout, well-being

## Abstract

**Background/Objectives:** Healthcare organizations are increasingly challenged by workforce shortages, occupational stress, burnout, moral distress, and staff turnover. Workplace spirituality has been associated with positive outcomes among healthcare professionals, including well-being, engagement, job satisfaction, and organizational commitment. However, existing research has largely focused on these associations, with less attention to the organizational and interpersonal mechanisms through which workplace spirituality may influence healthcare professionals’ experiences and workforce sustainability. This conceptual paper aims to develop an integrative framework explaining how workplace spirituality may contribute to healthcare professionals’ well-being through interconnected organizational and interpersonal processes. **Methods:** The paper synthesizes theoretical perspectives and empirical literature from workplace spirituality, spiritual leadership, psychosocial safety climate, psychological safety, meaningful work, Self-Determination Theory, and the Job Demands–Resources model. These perspectives are conceptually integrated to develop a multilevel framework linking workplace spirituality and spiritual leadership with psychosocial safety climate, psychological safety, and healthcare workforce outcomes. **Results:** The proposed framework conceptualizes workplace spirituality as a multidimensional, value-based psychosocial condition encompassing meaningful work, sense of community, and alignment between individual and organizational values. Spiritual leadership is positioned as an enabling mechanism through which these value-based principles may be translated into organizational priorities and practices. These processes are proposed to contribute to a positive psychosocial safety climate, reflecting organizational commitment to protecting employees’ psychological health. In turn, psychosocial safety climate may provide an organizational foundation for psychological safety within healthcare teams, facilitating open communication, speaking up, error reporting, help-seeking, learning, and adaptive coping. Psychological safety may subsequently contribute to improved well-being, resilience, engagement, and workforce retention, while reducing emotional exhaustion and burnout and potentially supporting patient safety. The strength of these proposed relationships may depend on contextual conditions such as professional hierarchy, workload, emotional demands, and organizational resources. **Conclusions:** The proposed framework offers a novel conceptual integration of workplace spirituality, spiritual leadership, psychosocial safety climate, and psychological safety into a multilevel explanatory pathway. Rather than treating workplace spirituality as a conventional organizational asset, the framework conceptualizes it as a value-based psychosocial condition that may enable the development and translation of relational and organizational resources. The framework provides a basis for future empirical research examining the proposed pathways, their directionality and boundary conditions, and their potential relevance to healthcare workforce well-being and sustainability.

## 1. Introduction

Healthcare professionals constitute the foundation of healthcare systems, playing a critical role in ensuring the accessibility, quality, and sustainability of healthcare services worldwide [1,2,3]. However, healthcare systems across the globe continue to experience persistent workforce shortages, defined as an insufficient supply of appropriately qualified professionals to meet the growing demand for healthcare services [1,2]. Population ageing, increasing prevalence of chronic disease, rising service complexity, and expanding healthcare needs have intensified demands on an already overstretched workforce, creating substantial challenges for healthcare organizations [1,3]. The COVID-19 pandemic further exposed longstanding vulnerabilities in workforce planning and human resource management, highlighting the indispensable role of healthcare professionals while simultaneously amplifying existing workforce pressures [4,5]. Consequently, ensuring a sustainable healthcare workforce has become a global priority for healthcare organizations, policymakers, and researchers alike.

The consequences of these workforce challenges extend well beyond staffing shortages. Healthcare professionals routinely work in highly demanding environments characterized by heavy workloads, time pressures, increasing administrative responsibilities, emotional labor, exposure to suffering and death, workplace violence, and growing expectations for high-quality, patient-centered care [6,7,8]. These cumulative occupational demands have been consistently associated with elevated levels of burnout, emotional exhaustion, compassion fatigue, moral distress, psychological distress, and turnover intentions across healthcare professions [9,10]. Burnout, in particular, has emerged as one of the most significant occupational challenges facing healthcare systems, compromising clinicians’ well-being while simultaneously affecting patient safety, quality of care, organizational performance, and workforce retention [11,12,13]. Although strategies such as improving staffing levels, redesigning work processes, expanding professional roles, resilience training, and leadership development remain essential, there is increasing recognition that structural solutions alone are unlikely to fully address the complex psychological and relational challenges experienced by healthcare professionals [14,15,16].

Against this backdrop, growing attention has been directed towards workplace conditions that foster meaning, connectedness, value congruence, and supportive relationships. One construct that has attracted increasing scholarly interest is workplace spirituality. Broadly conceptualized as employees’ experience of meaningful work, connectedness with others, alignment between personal and organizational values, and a sense of purpose within the workplace, workplace spirituality has been associated with numerous positive organizational and individual outcomes [17,18]. Within healthcare settings, studies suggest that workplace spirituality is associated with greater job satisfaction, organizational commitment, work engagement, resilience, psychological well-being, and lower levels of burnout and turnover intentions [19,20,21]. Given the inherently value-driven and relational nature of healthcare work, workplace spirituality has been increasingly proposed as a potentially important value-based psychosocial condition for supporting healthcare professionals in demanding clinical environments [19,20,21].

However, the term “organizational resource” requires conceptual clarification in the context of workplace spirituality. In this paper, workplace spirituality is not conceptualized as a tangible or intangible organizational asset possessed by the organization. Rather, it is conceptualized as an upstream, value-based psychosocial condition through which employees may experience meaning, belonging, value congruence, and relational connection. Its potential resource value lies in the psychological and relational resources it may generate, reinforce, or make accessible to employees, rather than in workplace spirituality being a discrete organizational asset in itself.

Despite this growing body of evidence, the literature remains dominated by studies demonstrating direct associations between workplace spirituality and desirable employee outcomes. Although these findings consistently suggest beneficial relationships, they provide limited insight into the underlying organizational and psychological processes through which workplace spirituality influences healthcare professionals’ experiences and well-being. Consequently, current evidence explains whether workplace spirituality is beneficial far more effectively than how or why these benefits emerge. Moreover, existing conceptualizations have largely focused on workplace spirituality as an individual-level experience, giving comparatively less attention to how organizational conditions and interpersonal workplace climates translate value-based experiences into sustainable improvements in employee well-being. Addressing these explanatory gaps is essential for advancing theory and informing interventions that move beyond promoting workplace spirituality as an abstract organizational ideal.

Workplace spirituality also requires conceptual differentiation from adjacent constructs. Although it overlaps with meaningful work, organizational identification, organizational culture, and work engagement, these constructs are not interchangeable. Workplace spirituality integrates experiences of meaningful work, sense of community, and alignment between individual and organizational values [17,18], whereas related constructs typically capture more specific psychological states, organizational processes, or workplace conditions. For example, work engagement concerns vigor, dedication, and absorption [22], while meaningful work focuses primarily on the perceived significance and purpose of one’s work [23]. These distinctions are important for positioning workplace spirituality within the proposed multilevel framework and are examined in greater detail in Section 2.

One promising mechanism through which workplace spirituality may influence healthcare professionals’ well-being is psychological safety. Originally introduced by Edmondson [24], psychological safety refers to employees’ shared belief that they can express ideas, ask questions, admit mistakes, raise concerns, and seek assistance without fear of embarrassment, punishment, or negative interpersonal consequences. Although previous research has identified leadership, social support, and organizational culture as important antecedents of psychological safety [25,26,27], less attention has been given to the broader value-based and organizational conditions through which psychologically safe environments emerge. Workplace spirituality may represent one such upstream condition because meaningful work, sense of community, and alignment between individual and organizational values may support relational qualities such as trust, mutual respect, openness, and connection. These qualities may, in turn, facilitate employees’ willingness to engage in interpersonal risk-taking. Integrating workplace spirituality with psychological safety therefore provides an opportunity to move beyond examining whether spirituality is beneficial and towards understanding how value-based workplace conditions may influence healthcare professionals’ capacity to communicate, learn, seek support, and cope with challenging experiences.

Furthermore, although psychological safety has received increasing attention within healthcare research, it primarily captures interpersonal experiences occurring within teams. Less consideration has been given to the broader organizational conditions that shape psychologically safe environments. In this respect, psychosocial safety climate (PSC) [28] provides an important complementary perspective by reflecting employees’ shared perceptions that organizational policies, leadership practices, and management priorities actively protect psychological health and minimize psychosocial risks. PSC therefore occupies a different conceptual level from workplace spirituality and psychological safety: whereas workplace spirituality concerns employees’ experiences of meaning, community, and values alignment, PSC concerns shared perceptions of organizational priorities and practices relating to psychological health, and psychological safety concerns the interpersonal experience of whether it is safe to take interpersonal risks within a team. Integrating PSC with workplace spirituality and psychological safety therefore enables a multilevel understanding of how value-based workplace conditions may be translated into organizational priorities and, ultimately, psychologically safe workplace experiences.

This multilevel perspective is particularly relevant to healthcare, where professional hierarchies, multidisciplinary interdependence, clinical uncertainty, moral distress, and high emotional demands can make open communication and help-seeking both essential and difficult [29,30]. Psychological safety enables healthcare professionals to speak up, question decisions, acknowledge uncertainty, report concerns, and learn from complex clinical situations, with implications for teamwork, learning, and the quality and safety of healthcare delivery [31,32]. In healthcare settings, these processes are embedded within broader ethical and psychosocial conditions [33]. Research among healthcare professionals has highlighted the importance of ethical values and moral responsibility within healthcare organizations [32], while empirical evidence has linked adverse workplace experiences with burnout, negative affect, poorer general health, and lower perceived group cohesion among nurses [34,35,36]. Such findings underscore the importance of organizational and interpersonal conditions that shape how healthcare professionals experience their work and interact with colleagues.

Accordingly, this conceptual paper develops an integrative multilevel framework explaining how workplace spirituality may contribute to healthcare professionals’ well-being through interconnected leadership, organizational, and interpersonal processes. Drawing upon workplace spirituality theory, psychosocial safety climate, psychological safety, spiritual leadership, Self-Determination Theory, and the Job Demands–Resources model, we propose that workplace spirituality functions as an upstream value-based psychosocial condition that may be translated through spiritual leadership into organizational priorities and practices supporting psychosocial safety climate. A positive psychosocial safety climate may subsequently provide an organizational foundation for psychological safety within healthcare teams, enabling healthcare professionals to engage in open communication, learning, adaptive coping, and help-seeking behaviors that may strengthen well-being while reducing burnout and emotional exhaustion.

By bringing together theoretical perspectives that have often been examined separately, this paper makes three related contributions. First, it shifts attention from documenting associations between workplace spirituality and employee outcomes towards explaining the organizational and interpersonal processes through which these associations may emerge. Second, it positions psychosocial safety climate as an organizational bridge between value-based workplace conditions and interpersonal psychological safety. Third, it conceptualizes spiritual leadership as an enabling mechanism through which value-based principles may be translated into leadership behaviors and organizational practices, while recognizing that workplace spirituality and spiritual leadership may also be mutually reinforcing in practice. The proposed framework therefore offers a theoretically grounded basis for future empirical research examining the directionality, mechanisms, and boundary conditions of these relationships and may inform healthcare organizations seeking to create psychologically healthy and sustainable workplaces.

The theoretical contribution of this paper does not rest on introducing a new construct or claiming that the individual relationships among workplace spirituality, spiritual leadership, psychosocial safety climate, psychological safety, and well-being have not previously been examined. Rather, the contribution lies in their theoretically specified integration across organizational and interpersonal levels. Existing workplace spirituality research has frequently examined direct associations with outcomes such as well-being, job satisfaction, engagement, commitment, and burnout, while mediation studies have commonly focused on individual-level psychological or attitudinal mechanisms. The present framework extends this literature by proposing that workplace spirituality may function as an upstream value-based psychosocial condition whose effects may be translated through leadership and organizational psychosocial conditions before becoming reflected in proximal interpersonal experiences of psychological safety and, ultimately, employee well-being.

A particularly important distinction concerns the role of PSC. PSC is not treated as another form of supportive leadership or as an alternative indicator of psychological safety. Rather, PSC captures shared perceptions of organizational management priorities, policies, and practices concerning the protection of employees’ psychological health. Its inclusion therefore provides an organizational-level mechanism through which value-based leadership and workplace conditions may become reflected in the broader psychosocial environment. Psychological safety operates at a more proximal interpersonal or team level, concerning employees’ perceptions of whether they can speak up, seek help, acknowledge uncertainty or mistakes, and express concerns without fear of interpersonal consequences. By connecting these levels, the framework seeks to explain how value-based workplace experiences may be translated into organizational and interpersonal conditions relevant to healthcare professionals’ well-being.

Accordingly, the proposed framework should be understood as a conceptual integration of established constructs rather than as a claim of an entirely new theory. Its added value lies in specifying a multilevel explanatory pathway and identifying theoretically meaningful points at which translation, reinforcement, or disruption may occur. This formulation also creates empirically testable questions concerning cross-level relationships, temporal ordering, reciprocal processes, and contextual boundary conditions. The framework is intended as a conceptual model that organizes theoretically plausible relationships rather than as an established causal model. Its principal theoretical contribution lies in addressing a gap between two bodies of literature that have often been considered separately: workplace spirituality research, which has frequently emphasized direct or individual-level associations with employee outcomes, and organizational psychosocial research, which has examined leadership, psychosocial safety climate, and psychological safety without explicitly incorporating workplace spirituality as a value-based upstream condition. The present framework connects these perspectives by specifying how value-based workplace experiences may be translated through leadership and organizational psychosocial conditions into proximal interpersonal processes relevant to employee well-being.

This distinction is important because the framework is not simply a rearrangement of existing constructs. Each component addresses a different explanatory level and mechanism: workplace spirituality represents the value-based psychosocial condition; spiritual leadership represents a leadership-level translation mechanism; PSC represents the organizational-level prioritization of psychological health; psychological safety represents the proximal interpersonal condition governing voice and interpersonal risk; and well-being represents the individual-level outcome. The proposed contribution is therefore the specification of a multilevel translation pathway that can be empirically distinguished from direct workplace spirituality effects, predominantly individual-level mediation models, and alternative leadership-centred pathways.

### Conceptual Framework Development Approach

This article uses a conceptual theory-building approach rather than a systematic or scoping review methodology. Accordingly, the objective was not to provide an exhaustive synthesis of all literature concerning workplace spirituality, leadership, psychosocial safety, or healthcare well-being. Instead, the framework was developed through an iterative conceptual synthesis of established theoretical and empirical literature selected for its relevance to the proposed explanatory problem: how value-based workplace experiences may become connected to healthcare professionals’ well-being through organizational and interpersonal mechanisms.

The selection of constructs was guided by two principles: conceptual relevance and non-redundant explanatory function. Five linked domains were therefore incorporated: workplace spirituality, spiritual leadership, psychosocial safety climate, psychological safety, and healthcare professionals’ well-being and workforce-related outcomes. These constructs were selected because they address different but potentially connected levels of the proposed process.

The theoretical perspectives used in the framework were selected according to their explanatory contribution rather than to maximize the number of theories included. Workplace spirituality provides the conceptual basis for understanding meaning, community, and alignment between individual and organizational values. Spiritual leadership provides a leadership perspective on how values may be enacted and translated into organizational practices. Psychosocial safety climate provides an organizational-level perspective on management priorities and practices concerning psychological health. Psychological safety provides an interpersonal perspective on voice, help-seeking, error disclosure, and interpersonal risk. Self-determination theory provides a complementary explanation for the motivational relevance of meaning, relatedness, and value congruence, whereas the Job Demands–Resources perspective situates these processes within broader workplace demands and resources.

The proposed relationships were established through integration of established construct definitions, prior theoretical arguments, empirical findings, and conceptual compatibility across levels of analysis. Importantly, the framework distinguishes between relationships supported by previous literature and relationships that constitute the authors’ proposed conceptual synthesis. The directional sequence presented in the framework therefore represents a theoretically specified pathway for future empirical testing rather than an established causal model. Alternative and reciprocal relationships were also considered and are discussed as important directions for future research.

In the following sections, the theoretical foundations and conceptual relationships underlying the proposed framework are examined in greater detail.

## 2. Workplace Spirituality as a Value-Based Psychosocial Condition

Over the past two decades, workplace spirituality has emerged as an important construct in assessments within organizational behavior and occupational health research. Although early discussions frequently associated spirituality with religion or faith-based organizations, contemporary conceptualizations distinguish workplace spirituality from religious belief and instead describe it as a secular organizational phenomenon concerned with employees’ experience of meaningful work, interpersonal connectedness, and alignment between personal and organizational values [37,38,39]. Rather than promoting particular belief systems, workplace spirituality reflects the extent to which employees perceive that their work has purpose, that they belong to a supportive community, and that organizational values are consistent with their own professional and ethical commitments.

Despite continuing debate regarding its conceptual boundaries and occasional overlap with related constructs such as meaningful work, organizational culture, ethical leadership, and organizational identification [40], workplace spirituality has become increasingly recognized as a value-based psychosocial condition with potential resource-generating or resource-enabling properties. This recognition reflects broader changes in contemporary workplaces, where employees increasingly seek authenticity, purpose, and psychological fulfilment alongside traditional extrinsic rewards [41]. Such needs are particularly salient within healthcare organizations, where professionals routinely encounter emotionally demanding situations, ethical dilemmas, patient suffering, and death [42]. Importantly, workplace spirituality should not be interpreted as an inherently positive organizational practice. Critics have raised concerns that spirituality initiatives may become problematic when organizations attempt to prescribe particular values, encourage emotional conformity, or use spiritual language to individualize problems that are fundamentally structural [43]. Therefore, the potential benefits of workplace spirituality depend on whether it is experienced as authentic, inclusive, and supportive rather than imposed or instrumentalized by organizations. Accordingly, its potential resource value is conditional rather than inherent: workplace spirituality is expected to function constructively when employees experience meaning, belonging, and value alignment as authentic features of the workplace rather than as organizationally imposed expectations.

A growing body of empirical evidence suggests that workplace spirituality is associated with a range of favorable organizational and employee outcomes. Across occupational settings, higher levels of workplace spirituality have been linked with greater job satisfaction, organizational commitment, work engagement, motivation, organizational citizenship behavior, and psychological well-being [44,45]. Recent systematic reviews similarly conclude that spiritually supportive workplaces are generally associated with improved resilience, emotional health, and positive workplace experiences, while also emphasizing that organizational spirituality should remain inclusive and should not be conflated with religious expression [20]. Nevertheless, although these findings consistently demonstrate beneficial associations, they remain largely descriptive and provide limited explanation of the mechanisms through which workplace spirituality influences employee outcomes.

Most conceptualizations converge around three interrelated dimensions originally proposed by Milliman et al. [38]: meaningful work, sense of community, and alignment between individual and organizational values. Meaningful work enables employees to perceive their work as contributing to purposes beyond routine task completion. Sense of community reflects interpersonal trust, belonging, and mutual support within the workplace. Values alignment refers to the congruence between employees’ personal values and those promoted by the organization. Collectively, these dimensions may function as sources of psychological and relational support, enabling employees to interpret demanding work environments more positively while maintaining motivation, professional identity, and emotional resilience.

These characteristics appear especially relevant within healthcare. Caring professions are inherently purpose-driven and relational, making meaning, connection, compassion, and professional values central features of professional identity [41,42]. Emerging evidence suggests that workplace spirituality may support healthcare professionals by strengthening resilience, reducing emotional exhaustion, enhancing organizational commitment, and promoting high-quality patient care through greater alignment between professional values and organizational culture [46]. Likewise, spiritual leadership, through its emphasis on vision, hope, altruistic love, and shared purpose, has been associated with improved nurse well-being, reduced emotional fatigue, greater work engagement, and enhanced organizational performance [47,48]. These findings suggest that workplace spirituality and spiritual leadership may be closely related but conceptually distinct: workplace spirituality concerns employees’ experiences of meaning, community, and value alignment, whereas spiritual leadership concerns leadership behaviors and practices through which shared purpose and relational values may be enacted.

However, an important limitation within this literature remains. Most empirical studies conceptualize workplace spirituality primarily as an independent predictor of desirable outcomes, demonstrating positive associations with well-being, engagement, job satisfaction, organizational commitment, and reduced turnover intentions [20,49]. Although these studies establish that workplace spirituality matters, they offer relatively limited insight into the organizational and psychological processes through which these outcomes emerge. Consequently, workplace spirituality is often treated as a predictor of employee outcomes without sufficiently examining the organizational processes through which its potential benefits may be realized.

Some recent studies have begun to conceptualize workplace spirituality as a mediating mechanism linking organizational practices to employee outcomes [49,50], reflecting an emerging shift from asking whether workplace spirituality influences employee well-being towards understanding how these effects occur. Nevertheless, this literature remains in its infancy, and comparatively little attention has been devoted to identifying the relational and organizational conditions through which spiritually supportive workplaces translate into improved psychological functioning among healthcare professionals. In particular, limited conceptual attention has been given to the possibility that workplace spirituality may operate through organizational and interpersonal conditions, rather than solely through direct effects on individual employee outcomes.

This limitation is particularly important in healthcare settings, where professionals routinely navigate uncertainty, emotionally challenging clinical situations, hierarchical organizational structures, and the possibility of making mistakes with serious consequences [29,30]. Under such conditions, it is unlikely that workplace spirituality influences well-being solely because employees experience greater meaning or stronger value congruence. Rather, these experiences may shape the broader interpersonal climate by fostering trust, openness, mutual respect, and relational connection, thereby creating conditions that enable employees to communicate openly, seek support, and learn from challenging experiences. These relational processes provide a conceptual link to psychological safety, although workplace spirituality and psychological safety remain distinct constructs.

Accordingly, this paper argues that psychological safety represents a theoretically plausible mechanism through which workplace spirituality may contribute to healthcare professionals’ well-being. Rather than conceptualizing workplace spirituality as exerting only direct effects on employee outcomes, the proposed framework suggests that its influence may be realized, at least partly, through organizational and relational processes that support psychologically safe work environments. The following section therefore examines psychological safety and considers its role as the proximal interpersonal mechanism linking workplace spirituality and healthcare professionals’ well-being.

Workplace spirituality should also be distinguished from related constructs with which it may conceptually overlap. Meaningful work captures the experience that one’s work is purposeful and significant [23], whereas workplace spirituality incorporates meaningfulness within a broader framework that also emphasizes connection with others and alignment between individual and organizational values [17,30]. Organizational identification concerns the extent to which individuals define themselves in terms of organizational membership [40], while workplace spirituality concerns the experience of meaning, community, and value congruence within work [17,30]. Similarly, organizational culture refers to shared assumptions, norms, and values within an organization [25,26,27], whereas workplace spirituality focuses more specifically on employees’ experiences of meaning, connection, and value alignment [17,30]. Ethical leadership and compassion may contribute to spiritually supportive environments, but they represent leadership behaviors or relational qualities rather than dimensions of workplace spirituality itself [40]. Psychological safety and psychosocial safety climate are likewise conceptually distinct: psychological safety concerns perceptions of interpersonal risk within teams, whereas psychosocial safety climate concerns shared perceptions of organizational priorities, policies, and practices concerning psychological health [17,18,25].

Accordingly, this paper does not conceptualize workplace spirituality as a higher-order construct that subsumes these related concepts. Rather, workplace spirituality is treated as a multidimensional construct with three established dimensions: meaningful work, sense of community, and alignment between individual and organizational values [38]. Its distinctive contribution to the present framework is therefore not that it introduces entirely unique psychological experiences, but that it integrates these dimensions around the broader experience of purpose, connection, and value congruence within working life. This multidimensional conceptualization also provides a basis for examining how employees’ value-based workplace experiences may connect with leadership practices, organizational psychosocial conditions, and interpersonal psychological safety.

## 3. Psychological Safety as a Mechanism Linking Workplace Spirituality and Healthcare Professionals’ Well-Being

Psychological safety has become one of the most influential concepts for understanding employee behavior, learning, and well-being within complex organizational environments. Rather than reflecting individual confidence alone, psychological safety represents an important characteristic of workplace relationships and organizational culture that enables constructive interpersonal risk-taking [24]. While psychological safety is classically conceptualized as a shared team-level belief, it is also frequently assessed through individual employees’ perceptions of interpersonal risk within their teams. In contrast, psychosocial safety climate reflects broader organizational priorities and systems related to protecting psychological health [18,51]. Distinguishing these concepts is essential because organizational commitment to psychological health does not automatically guarantee that employees experience their immediate team environment as safe.

Unlike traditional notions of workplace safety, which primarily concern physical hazards and accident prevention, psychological safety focuses on the social and interpersonal conditions that enable employees to participate fully within organizations [52]. It reflects whether employees perceive that their workplace supports openness, respect, inclusion, and honest communication. This distinction is particularly important within healthcare, where safe patient care depends not only on technical competence but also on effective teamwork, continuous learning, and willingness to speak up when concerns arise [29,30].

Healthcare professionals operate within complex systems characterized by heavy workloads, emotional labor, uncertainty, frequent exposure to adverse events, and hierarchical professional structures [53]. Within these environments, psychological safety becomes particularly important because high-quality care depends upon employees’ willingness to communicate concerns, question decisions, report mistakes, seek assistance, and share knowledge. When psychological safety is lacking, professionals may remain silent because of fear of blame, reputational damage, or negative career consequences. Such silence limits learning, weakens teamwork, and may compromise both patient safety and employee well-being [24,54].

Consistent evidence demonstrates that psychologically safe workplaces are associated with healthier organizational outcomes. Employees who perceive higher psychological safety report stronger trust, greater work engagement, better collaboration, and lower burnout and turnover intentions [55,56]. Within healthcare, psychological safety has increasingly been recognized as an important protective psychosocial condition that may reduce exposure to occupational stress and emotional exhaustion. For example, Wang et al. [57] demonstrated that psychological safety mediated the relationship between servant leadership and burnout, suggesting that supportive leadership reduces psychological strain by creating environments where employees feel respected and protected.

More recent evidence further reinforces this protective role. A scoping review by Sato et al. [58] identified a consistent inverse relationship between psychological safety and burnout among nurses, concluding that psychologically safe work environments may have a protective role, particularly within high-intensity clinical settings. Similarly, Cogan et al. [59] demonstrated that both individual and team psychological safety were associated with lower burnout and acute stress among health and social care workers, with team-level psychological safety showing particularly strong associations with workforce well-being. Collectively, these findings suggest that psychological safety is not simply beneficial for communication and learning but also represents an important determinant of healthcare workforce sustainability.

Existing literature has primarily conceptualized psychological safety as a consequence of leadership, communication practices, and team dynamics. Inclusive leadership, supportive interpersonal relationships, trust, employee voice, and non-punitive responses to mistakes have consistently been identified as important antecedents of psychological safety [40,54]. Importantly, psychological safety should not be interpreted as the absence of disagreement or conflict. Rather, psychologically safe environments encourage constructive challenge, questioning, and learning because employees believe that differing opinions and honest discussion will be treated respectfully.

While these antecedents are well established, comparatively less attention has been devoted to the broader organizational values that make psychologically safe environments possible. This represents an important theoretical gap because leadership behaviors and communication practices rarely develop independently of the organizational values that shape them. Workplace spirituality may therefore provide an important upstream value-based psychosocial context within which psychological safety develops.

Conceptually, workplace spirituality and psychological safety share several underlying relational principles. Meaningful work reinforces employees’ perceptions that their contributions are valued [59]; sense of community strengthens interpersonal trust and belonging [60]; and values alignment promotes authenticity, integrity, and mutual respect [61]. Together, these characteristics may create organizational environments in which employees feel accepted, supported, and psychologically secure. In this way, workplace spirituality may not influence employee well-being directly alone, but indirectly by strengthening the interpersonal conditions necessary for psychological safety to emerge.

Emerging empirical evidence provides preliminary support for this proposition. Pahuja et al. [18] found that dimensions of workplace spirituality significantly predicted psychosocial safety climate, providing preliminary empirical evidence consistent with the proposed association between workplace spirituality and PSC. However, this finding does not establish that workplace spirituality necessarily produces positive PSC, nor does it by itself establish the sequential pathway proposed in this paper. Similarly, workplace spirituality may strengthen positive emotional experiences such as gratitude, hope, and compassion, which according to broaden-and-build theory [61,62] expand individuals’ psychological resources and strengthen interpersonal relationships during periods of stress. Within healthcare, where professionals frequently encounter emotionally demanding situations and moral challenges, these positive relational resources may provide an important foundation for psychological safety.

Taken together, the existing literature suggests that workplace spirituality, psychosocial safety climate, and psychological safety should not be viewed as independent organizational constructs but as conceptually distinct and potentially interconnected processes operating at different levels of organizational functioning. Workplace spirituality provides a value-based context characterized by meaningful work, sense of community, and alignment between individual and organizational values; spiritual leadership may translate these values into leadership behaviors and organizational practices; psychosocial safety climate reflects shared perceptions that organizational systems and leadership prioritize psychological health; and psychological safety reflects the proximal interpersonal experience that employees can take interpersonal risks without fear of negative consequences (Table 1). Building on this integration, the following sections propose a multilevel conceptual framework explaining how workplace spirituality may contribute to healthcare professionals’ well-being through the proposed sequential development of supportive organizational and interpersonal conditions.

These relationships may be particularly consequential in healthcare because clinical work combines high emotional demands with professional hierarchies, multidisciplinary interdependence, clinical uncertainty, and substantial responsibility for patient safety [29,30]. Hierarchical structures may make it difficult for junior or less powerful professionals to question decisions or disclose concerns, while multidisciplinary care requires frequent communication across professional boundaries [31]. Similarly, exposure to suffering, ethical conflict, adverse events, and situations in which available resources do not align with professional values may challenge healthcare professionals’ sense of meaning and contribute to moral distress. Within this context, value-based organizational conditions may be particularly relevant when they are translated into leadership practices and psychosocial climates that legitimize speaking up, seeking assistance, acknowledging uncertainty, and learning from adverse events. Thus, the healthcare context is not merely the empirical setting for the proposed framework but provides a theoretically important context in which the proposed mechanisms may become especially salient.

## 4. Integrating Workplace Spirituality, Psychosocial Safety Climate, and Psychological Safety: A Multilevel Conceptual Framework

The proposed framework was developed through an integrative synthesis of theoretical perspectives on workplace spirituality, spiritual leadership, psychosocial safety climate, psychological safety, self-determination, and job demands–resources. Rather than proposing a new standalone theory, the framework brings together established constructs that have generally been examined separately and specifies their potential relationships across different organizational levels. Workplace spirituality theory [38], spiritual leadership theory [63], psychosocial safety climate [64], psychological safety [24], self-determination theory [65], and the Job Demands–Resources model [66] are therefore incorporated according to their explanatory roles. This integration enables the framework to distinguish between the value-based experience represented by workplace spirituality, the leadership processes through which such values may be enacted, the organizational prioritization of psychological health represented by psychosocial safety climate, and the proximal interpersonal condition represented by psychological safety.

### 4.1. Workplace Spirituality as a Value-Based Psychosocial Condition in Healthcare

The Job Demands–Resources (JD-R) model provides a useful complementary perspective for understanding how value-based psychosocial conditions may influence healthcare professionals’ responses to demanding work environments. According to the JD-R model, job demands refer to physical, psychological, social, or organizational aspects of work that require sustained effort and may contribute to strain, whereas job resources help employees achieve work goals, reduce the impact of demands, and stimulate motivation [66]. In healthcare settings, workload, emotional demands, exposure to traumatic events, and interpersonal conflict represent significant job demands, while resources such as autonomy, social support, learning opportunities, and supportive relationships can buffer their negative effects [67]. From this perspective, workplace spirituality is not necessarily conceptualized as a conventional job resource such as autonomy, feedback, or staffing. Rather, it may represent a broader value-based psychosocial condition that can support the availability, interpretation, and use of psychological and relational resources by fostering meaningful work, interpersonal connection, and alignment between personal and organizational values.

Within this perspective, workplace spirituality concerns the extent to which employees perceive their work as purposeful, experience a sense of community, and recognize consistency between their own values and those promoted by the organization [37,38]. This perspective is particularly relevant in healthcare because many professionals are motivated by values of caring, service, and contribution to human well-being. When organizational pressures and administrative demands create distance between healthcare professionals and the meaningful aspects of their profession, frustration, moral distress, and emotional exhaustion may increase.

Qualitative evidence among nurses further highlights the importance of workplace spirituality in sustaining professional meaning. Habibpour [68] found that nurses understood workplace spirituality through themes including human-centeredness, moral values, and spiritual coping strategies. Although challenging working conditions and ethical conflicts could undermine perceptions of a spiritually supportive workplace, nurses attempted to preserve meaning through reflection, self-care, and meaning reconstruction. These findings suggest that workplace spirituality may represent an important value-based psychosocial condition shaping how healthcare professionals interpret workplace challenges and maintain psychological resilience.

However, workplace spirituality should not be understood as an inherently positive organizational practice. Concerns have been raised that spirituality initiatives may become problematic when organizations attempt to impose particular values, encourage emotional conformity, or shift responsibility for systemic problems onto individual employees [20,65]. Therefore, the potential benefits of workplace spirituality depend on whether it is experienced as authentic, inclusive, and supportive rather than as an organizational strategy used to compensate for inadequate working conditions. This distinction is particularly important in healthcare environments, where meaningful work must coexist with appropriate organizational resources and protections.

Accordingly, this paper does not conceptualize workplace spirituality as a higher-order construct that subsumes these related concepts. Rather, workplace spirituality is treated as a multidimensional construct with three established dimensions: meaningful work, sense of community, and alignment between individual and organizational values [38]. Its distinctive contribution to the present framework is therefore not that it introduces entirely unique psychological experiences, but that it brings these dimensions together around the broader question of how individuals experience purpose, connection, and value congruence in their working lives.

### 4.2. Spirituality and Psychological Need Fulfilment: A Self-Determination Theory Perspective

The relationship between workplace spirituality and healthcare professionals’ well-being can also be explained through Self-Determination Theory (SDT) [65]. SDT proposes that individuals possess three fundamental psychological needs: autonomy, competence, and relatedness. The fulfilment of these needs promotes intrinsic motivation, psychological growth, and well-being, whereas environments that frustrate these needs contribute to disengagement and psychological distress [69,70].

Workplace spirituality shares important conceptual similarities with SDT because both perspectives emphasize meaningful experiences, personal values, and supportive relationships as essential conditions for positive functioning. Workplace spirituality may support autonomy by enabling employees to perceive their work as personally meaningful and consistent with their values. This is particularly important in healthcare, where professional identity is frequently connected with caring and service-oriented motivations [41,42]. When healthcare professionals experience their work as purposeful rather than merely task-based, they may maintain stronger motivation and commitment despite demanding circumstances.

Workplace spirituality may also enhance competence by creating environments where employees feel recognized, valued, and supported in their professional development. Opportunities for learning, appreciation of contributions, and constructive feedback can strengthen healthcare professionals’ confidence in their abilities and their perception that their work has value. Furthermore, the community dimension of workplace spirituality reflects the SDT concept of relatedness by promoting interpersonal connection, trust, and belonging. In emotionally demanding healthcare environments, supportive relationships with colleagues and leaders can provide essential protection against isolation and psychological strain [71].

Recent healthcare research supports the importance of psychological need fulfilment for workforce well-being. Sanseverino et al. [72] demonstrated that supportive leadership relationships influenced healthcare professionals’ job satisfaction through mechanisms including autonomy, team cohesion, and opportunities for professional development. These findings reinforce the argument that organizational environments supporting fundamental psychological needs contribute to positive occupational outcomes. Therefore, workplace spirituality may provide a value-based foundation through which organizations create conditions that satisfy employees’ psychological needs.

### 4.3. Spiritual Leadership as an Enabling Mechanism

Although workplace spirituality concerns employees’ experiences of meaning, belonging, and value alignment, these experiences do not develop independently from organizational structures and leadership practices. Spiritual leadership provides an important mechanism through which value-based principles associated with workplace spirituality may become embedded within organizational culture and everyday workplace interactions.

Spiritual leadership is a values-based approach that emphasizes shared purpose, compassion, service, and the development of meaningful relationships between leaders and employees. Fry et al. [63] describe spiritual leadership as a framework that motivates employees through shared vision and a culture based on altruistic values. Central elements include calling, whereby employees perceive their work as meaningful and significant, and membership, whereby employees experience belonging and connection within the organization [63].

These principles are highly relevant within healthcare organizations, where professionals often seek alignment between their personal commitment to caring and the values demonstrated by their workplace. Leaders who demonstrate compassion, humility, integrity, and genuine concern for employees communicate that individuals are valued beyond their technical contributions [73]. Such leadership behaviors may strengthen trust, reduce fear, and create conditions where employees feel supported and respected.

Importantly, the relationship between spiritual leadership and workplace spirituality may be reciprocal in organizational practice. Spiritual leadership may cultivate employees’ experiences of meaning, belonging, and value alignment by embedding shared purpose and relational values within organizational practices, while existing perceptions of workplace spirituality may shape how employees interpret and respond to leadership behaviors. However, for conceptual and explanatory purposes, the present framework specifies workplace spirituality as the upstream value-based psychosocial condition and spiritual leadership as a translating or enabling mechanism through which these values may become reflected in organizational practices and priorities. This directional specification does not imply that reciprocal or alternative relationships are absent; rather, it provides a theoretically parsimonious pathway that can be examined empirically in future longitudinal research.

Through these processes, spiritual leadership may contribute to psychologically healthy organizational climates by influencing how employees interpret organizational priorities. When leaders consistently demonstrate care, ethical responsibility, and openness, employees may perceive that their psychological well-being is a genuine organizational priority. Accordingly, spiritual leadership is positioned in the present framework as a translating mechanism through which value-based principles associated with workplace spirituality may become reflected in organizational practices and broader psychosocial conditions, including psychosocial safety climate.

### 4.4. From Workplace Spirituality to Psychosocial Safety Climate and Psychological Safety

Although workplace spirituality has been associated with positive employee outcomes, the organizational mechanisms explaining these relationships remain insufficiently developed. This paper proposes that PSC represents an important organizational-level mechanism linking workplace spirituality with psychological safety and, ultimately, healthcare professionals’ well-being.

PSC refers to employees’ shared perceptions regarding the extent to which organizational policies, practices, and leadership priorities protect psychological health and prevent psychosocial harm [64]. While workplace spirituality primarily reflects employees’ experiences of meaning, connection, and value congruence, PSC represents a broader organizational climate concerning whether psychological health is genuinely prioritized through organizational policies, management practices, leadership behaviors, and approaches to psychosocial risk. Accordingly, workplace spirituality and PSC are conceptually distinct: workplace spirituality concerns the meaning, belonging, and value-based experience of work, whereas PSC concerns the organizational priority given to psychological health.

Workplace spirituality may contribute to PSC by shaping organizational values and interpersonal expectations. Organizations that promote compassion, respect, ethical responsibility, and meaningful contribution may create environments where employees perceive that psychological health is valued. Such cultures may encourage supportive policies, open communication, employee involvement, and proactive responses to psychosocial risks. In this sense, workplace spirituality is not proposed to constitute PSC itself; rather, its value-based characteristics may provide a cultural and relational foundation from which organizational practices supportive of psychological health can develop. In healthcare settings, where professionals frequently experience emotional demands, workload pressures, and ethical challenges [9,10], these organizational conditions may be particularly important for sustaining workforce resilience.

The proposed relationship is therefore contingent on the extent to which value-based principles are translated into organizational practices. Workplace spirituality is unlikely to strengthen PSC merely because employees personally experience their work as meaningful. Rather, the expected relationship should be stronger when organizational leaders and systems consistently enact values such as respect, compassion, fairness, inclusion, and concern for employee well-being. Conversely, where organizational rhetoric emphasizes meaning or shared values but working conditions remain punitive, unsupportive, or indifferent to psychosocial risks, workplace spirituality may have limited capacity to generate a positive PSC.

A positive PSC may subsequently facilitate psychological safety. Although related, these concepts operate at different levels. PSC reflects shared organizational perceptions regarding policies, leadership priorities, and systems for protecting psychological health, whereas psychological safety reflects employees’ perceptions that they can express concerns, ask questions, share ideas, and acknowledge mistakes without fear of negative consequences [27]. Thus, PSC provides an organizational context that may support the development of psychological safety within teams and everyday interactions.

The proposed relationship can be understood through an organizational-to-interpersonal translation process. When organizational policies and leadership practices consistently communicate that psychological health is valued, employees may infer that speaking up, disclosing difficulties, asking for assistance, and reporting mistakes will be treated as legitimate rather than threatening behaviors. These organizational signals may reduce anticipated interpersonal and career-related risks and thereby strengthen psychological safety within teams. However, PSC should not be regarded as a sufficient condition for psychological safety, because team leadership, interpersonal relationships, professional hierarchies, and local team norms may strengthen or weaken the extent to which organizational priorities are experienced as psychologically safe in everyday interactions [18].

Healthcare research increasingly highlights the protective role of psychological safety. O’Donovan et al. [54] demonstrated that psychological safety within healthcare teams was influenced by leadership behaviors, interpersonal relationships, and perceptions of whether concerns would be valued and appropriately addressed. Similarly, Sato et al. [58] identified a consistent inverse relationship between psychological safety and burnout among nurses, suggesting that psychologically safe environments may have a protective role. Cogan et al. [59] further demonstrated that individual and team psychological safety were associated with well-being, burnout, and acute stress among health and social care workers, with team psychological safety showing particularly strong associations with burnout reduction.

These findings suggest that psychological safety may represent the proximal mechanism through which broader organizational conditions influence healthcare professionals’ psychological functioning. When employees feel able to speak up, seek help, acknowledge uncertainty, and discuss errors without fear of interpersonal punishment, they may be better able to mobilize social support, learn from difficult experiences, regulate occupational demands, and participate in adaptive problem-solving. The potential protective effect of psychological safety may therefore be particularly pronounced in high-demand healthcare environments where silence, concealment of difficulties, or reluctance to seek assistance can intensify occupational strain.

Together, these findings suggest that workplace spirituality may influence healthcare professionals’ well-being through a multilevel pathway. Workplace spirituality provides a value-based context characterized by meaningful work, sense of community, and alignment between individual and organizational values; these principles may be translated through spiritual leadership and organizational practices into a positive psychosocial safety climate; PSC may subsequently provide organizational conditions conducive to psychological safety within healthcare teams; and psychological safety may enable voice, help-seeking, learning, and constructive communication that support well-being and reduce occupational strain.

Importantly, this pathway does not imply that workplace spirituality is the sole or direct cause of PSC or psychological safety. Rather, it represents an upstream value-based condition that may complement established determinants of psychological safety, including supportive leadership, trust, team relationships, and non-punitive responses to error [52,54]. The proposed contribution is therefore to identify a less explicitly integrated in prior literature pathway through which workplace spirituality may become translated into organizational and interpersonal conditions that support healthcare professionals’ well-being.

Although preliminary empirical evidence has linked workplace spirituality with PSC [18], the present framework does not assume that meaningful work, sense of community, or value alignment automatically produce perceptions that psychological health is prioritized by management. Rather, the proposed relationship depends on organizational translation. These value-based experiences are more likely to contribute to positive PSC when organizational leaders and systems consistently enact values through supportive policies, fair decision-making, employee participation, attention to psychological health, and non-punitive responses to concerns. Where such organizational enactment is absent, workplace spirituality may remain primarily an individual experience without producing a corresponding perception of organizational commitment to psychological health.

### 4.5. Multilevel Conceptual Framework and Propositions

Building on the theoretical integration described above, this section specifies the proposed multilevel framework and the mechanisms through which workplace spirituality may contribute to healthcare professionals’ well-being. The framework is intended as a conceptual model that organizes theoretically plausible relationships rather than as an established causal model. Its originality therefore lies primarily in the integration of previously developed perspectives into a multilevel explanatory pathway linking value-based workplace experiences with organizational climate, interpersonal processes, and healthcare workforce outcomes.

For conceptual consistency, the framework adopts the three-dimensional formulation of workplace spirituality proposed by Milliman et al. [38]: meaningful work, sense of community, and alignment between individual and organizational values. Although compassion is relevant to spiritually supportive healthcare environments and is incorporated into the discussion of spiritual leadership and relational climate, it is not treated as a fourth constituent dimension of workplace spirituality in the present framework. This decision preserves conceptual continuity with the established three-dimensional formulation and avoids introducing an additional dimension without sufficient theoretical and empirical justification.

Although workplace spirituality and spiritual leadership may be mutually reinforcing in organizational practice, the present framework specifies a directional relationship for conceptual and explanatory purposes. Workplace spirituality is positioned as an upstream value-based psychosocial condition reflecting meaningful work, sense of community, and alignment between individual and organizational values, whereas spiritual leadership is conceptualized as a leadership-based mechanism through which these values may be translated into organizational practices and priorities [63]. Thus, the framework does not assume that spiritual leadership is an antecedent of workplace spirituality in all circumstances. Rather, it proposes that, within the specific explanatory pathway developed in this paper, spiritual leadership helps translate value-based experiences and principles into organizational conditions that support psychosocial safety climate. Reciprocal relationships may subsequently emerge and should be examined empirically in future longitudinal research.

**Figure 1:**
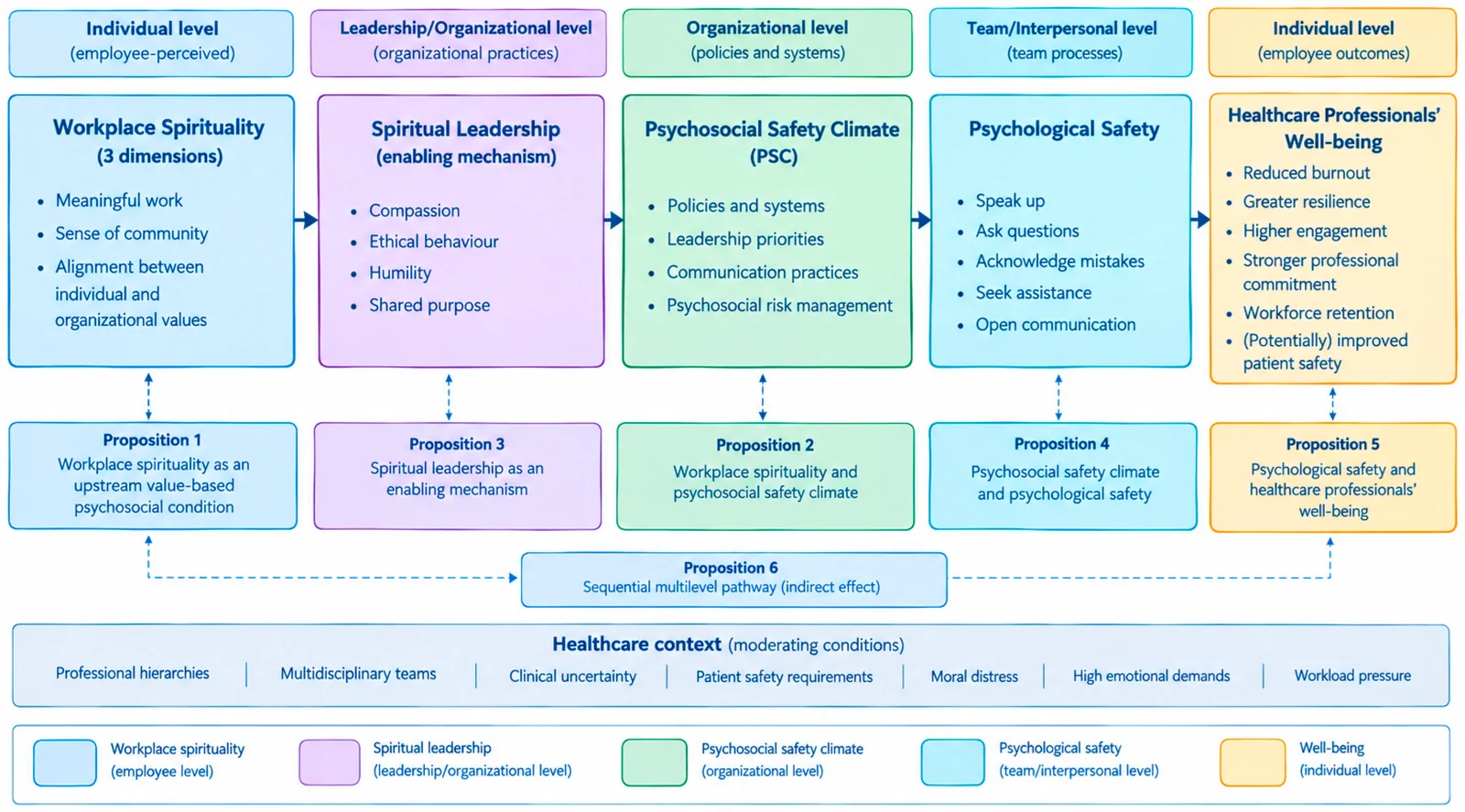
Proposed multilevel conceptual pathway linking workplace spirituality to healthcare professionals’ well-being.

The framework is presented in Figure 1 and should be read as a multilevel explanatory pathway rather than as a simple linear causal model. At the upstream level, workplace spirituality represents a value-based psychosocial condition characterized by meaningful work, sense of community, and alignment between individual and organizational values. These experiences may provide employees with meaning, belonging, and value congruence and may create a foundation for values-based leadership and organizational practices. Spiritual leadership is positioned as an enabling mechanism through which these values may be enacted in organizational life.

The framework is explicitly multilevel because the constructs occupy different but interconnected levels of analysis. Workplace spirituality is primarily conceptualized as an employee-perceived experience, although aggregation to team or organizational levels may be appropriate when sufficient within-group agreement is demonstrated. Spiritual leadership represents leadership behaviors and practices that may operate at the leadership or organizational level. PSC is principally an organizational-level construct reflecting shared perceptions of management priorities, policies, and practices concerning psychological health. Psychological safety is more proximal and is typically experienced within teams or interpersonal work relationships, whereas well-being and related workforce outcomes are primarily individual-level outcomes.

The ordering begins with workplace spirituality and proceeds through spiritual leadership, PSC, and psychological safety before reaching healthcare professionals’ well-being. This sequence represents the focal theoretical model rather than an assertion that these relationships are necessarily linear or unidirectional. The proposed cross-level relationships therefore require empirical attention to both aggregation and contextual translation. In particular, organizational PSC does not automatically produce psychological safety for every employee. Its influence may depend on proximal leadership, team norms, interpersonal trust, professional hierarchy, and opportunities for voice. Similarly, workplace spirituality should not be assumed to operate at an organizational level unless empirical evidence demonstrates sufficient within-group agreement. These considerations are important for future multilevel tests of the framework.

At the organizational level, these value-based leadership and organizational practices are proposed to contribute to psychosocial safety climate. PSC reflects the extent to which psychological health is prioritized through organizational policies, leadership behaviors, communication practices, and approaches to psychosocial risk [18,28]. The proposed relationship is expected to be stronger when organizational values are consistently enacted through supportive and non-punitive practices and weaker when organizational rhetoric is inconsistent with employees’ actual working conditions.

At the team and interpersonal level, psychological safety enables healthcare professionals to engage in behaviors essential for both workforce well-being and patient safety, including speaking up about concerns, reporting errors, seeking support, and participating in shared learning [24,54]. PSC is proposed to provide the organizational context in which these psychologically safe interactions are more likely to emerge, while team leadership and interpersonal relationships may strengthen or weaken this translation process.

At the individual level, these processes may contribute to reduced burnout, improved resilience, stronger professional functioning, and enhanced well-being. The framework therefore distinguishes between the organizational conditions that support psychological safety, the interpersonal behaviors enabled by psychological safety, and the individual outcomes that may result from experiencing such conditions.

The framework also distinguishes between the conceptual level of each construct and the level at which it may be empirically assessed. Workplace spirituality is primarily conceptualized as an employee-perceived experience of meaningful work, community, and value alignment, although shared perceptions may emerge within organizational units and may be aggregated when appropriate empirical agreement is established. Spiritual leadership concerns leadership behaviors and organizationally situated practices; psychosocial safety climate is conceptualized at the organizational level as shared perceptions that psychological health is protected and prioritized; and psychological safety represents a more proximal team-level or interpersonal perception concerning whether individuals can speak up, ask questions, acknowledge uncertainty, and discuss mistakes without fear of interpersonal or professional consequences. Workforce well-being and burnout are primarily individual-level outcomes.

Within this multilevel structure, PSC provides the organizational-to-team link in the proposed framework. Organizational policies, leadership priorities, and practices that demonstrate genuine concern for psychological health may create a context in which speaking up, help-seeking, and error discussion are legitimized. However, PSC is not assumed to automatically produce psychological safety at the team level. The translation from organizational climate to psychological safety may depend on local leadership, interpersonal trust, team norms, professional hierarchy, and the consistency with which organizational priorities are enacted in everyday interactions.

The framework therefore proposes the following mechanisms and boundary conditions:

**Proposition** **1.** *Workplace spirituality as an upstream value-based psychosocial condition.*

Workplace spirituality is proposed as an upstream value-based psychosocial condition that may shape how healthcare professionals experience their work and workplace relationships. Higher levels of meaningful work, sense of community, and alignment between individual and organizational values are expected to be associated with more favorable downstream psychosocial conditions, whereas imposed, exclusionary, or inconsistently enacted forms of spirituality may weaken or complicate these relationships.

**Proposition** **2.** *Workplace spirituality and psychosocial safety climate.*

Workplace spirituality is proposed to contribute to a positive psychosocial safety climate when its underlying values are translated into organizational priorities, policies, leadership practices, communication, and resource decisions that demonstrate genuine concern for employees’ psychological health. This relationship is expected to be stronger when organizational values are consistently enacted and weaker when they remain primarily symbolic or rhetorical [43,64].

**Proposition** **3.** *Spiritual leadership as an enabling mechanism.*

Spiritual leadership is proposed to function as a leadership-level translating mechanism through which workplace spirituality may become associated with psychosocial safety climate. Specifically, workplace spirituality may provide value-based experiences and expectations concerning meaning, community, and value alignment, while spiritual leadership may translate these principles into leadership behaviours, organizational priorities, and practices relevant to psychological health. This indirect relationship is expected to be stronger when leaders have sufficient authority, organizational resources, and opportunities to enact shared values, and weaker when excessive workload, punitive systems, structural constraints, or competing organizational priorities limit the enactment of these values.

**Proposition** **4.** *Psychosocial safety climate and psychological safety.*

PSC is proposed to facilitate psychological safety by establishing organizational conditions in which psychological health is prioritized and employees are more likely to perceive that speaking up, acknowledging mistakes, asking for assistance, and raising concerns will be supported [24,52,54]. However, the strength of this relationship is expected to depend on proximal team conditions, including supportive leadership, interpersonal trust, non-punitive responses to error, inclusive communication, and the configuration of professional hierarchy.

**Proposition** **5.** *Psychological safety and healthcare professionals’ well-being.*

Psychological safety is proposed to contribute to healthcare professionals’ well-being by facilitating voice, help-seeking, error disclosure, knowledge sharing, and interpersonal learning. These processes may reduce the interpersonal burden associated with remaining silent or concealing concerns and may therefore be associated with lower burnout and emotional exhaustion and with more favorable well-being outcomes [56,57,58,59]. The relevance of psychological safety is expected to be particularly pronounced in healthcare environments characterized by high workload, emotional demands, uncertainty, and hierarchical relationships.

**Proposition** **6.** *Sequential multilevel pathway.*

Workplace spirituality is expected to show an indirect association with healthcare professionals’ well-being through a sequential pathway involving spiritual leadership, psychosocial safety climate, and psychological safety, with the strength of this pathway expected to vary according to contextual conditions such as authenticity and consistency of organizational values, leadership support, workload, professional hierarchy, and available organizational resources. This proposed indirect pathway represents a hypothesis for future empirical testing rather than an established mediation effect.

The propositions are intended to provide a basis for empirical testing rather than to represent established causal relationships. Because the framework spans individual, team/interpersonal, leadership, and organizational levels, future studies should align measurement and analysis with the level at which each construct is theoretically located. Table 2 provides illustrative operational guidance for testing the proposed relationships. The measures listed are candidate approaches rather than prescribed instruments, and researchers should select validated measures appropriate to the healthcare population, cultural context, and level of analysis.

Taken together, these propositions specify a theoretically plausible sequence rather than established causal relationships: workplace spirituality provides an upstream value-based psychosocial condition; spiritual leadership may translate relevant values into leadership behaviors and organizational practices; these practices may contribute to a positive psychosocial safety climate; PSC may create organizational conditions conducive to psychological safety within teams; and psychological safety may enable behaviors that support healthcare professionals’ well-being. The framework therefore conceptualizes workplace spirituality as a value-based psychosocial condition with potential resource-generating or resource-enabling properties, rather than as a conventional organizational asset or a substitute for structural resources.

Importantly, the propositions do not imply that these relationships are deterministic or exclusive. Established determinants of psychological safety, including supportive leadership, interpersonal trust, team relationships, and non-punitive responses to error, remain relevant [52,54]. Similarly, workplace spirituality may itself be influenced by leadership, team experiences, and employee well-being over time. The proposed direction is therefore a theoretically specified pathway for empirical examination rather than a claim that alternative or reciprocal processes do not exist. Similarly, the proposed WPS → SL → PSC → psychological safety → well-being sequence should not be interpreted as the only possible explanation of the relationships among these constructs.

## 5. Practical Implications

The proposed framework has important implications for healthcare leaders, managers, and organizational policymakers seeking to promote sustainable workforce well-being. While workplace spirituality has traditionally been discussed primarily as an individual experience of meaning, connection, and personal fulfilment [37,38,39], the present framework suggests that its organizational relevance lies in the extent to which these experiences are supported and translated into everyday leadership practices, workplace systems, and relational processes. Accordingly, workplace spirituality should not be treated as a discrete organizational asset or as a substitute for adequate staffing, fair working conditions, or other structural resources. Rather, it may function as a value-based psychosocial condition that helps shape the relational and organizational environments in which healthcare professionals work.

Importantly, healthcare organizations should not attempt to promote spirituality through symbolic statements, isolated initiatives, or abstract organizational values. Instead, the framework suggests that the potential benefits of workplace spirituality depend on the consistency between organizational values and employees’ everyday experiences. Meaning, belonging, and value alignment are more likely to contribute to positive outcomes when they are reflected in leadership behavior, organizational policies, and practices that demonstrate respect, compassion, fairness, inclusion, and genuine concern for psychological health [74]. Conversely, symbolic or imposed forms of spirituality may have limited value, particularly when they coexist with punitive management, excessive workload, or inadequate organizational support.

A first implication concerns leadership development. Because spiritual leadership represents a key mechanism through which value-based principles may be translated into organizational practices [63], healthcare organizations should support leadership approaches characterized by compassion, ethical integrity, humility, and shared purpose. Leaders who actively listen, acknowledge employee contributions, respond constructively to concerns, and demonstrate genuine concern for staff well-being may help translate workplace spirituality into a psychosocial safety climate by signaling that psychological health is an organizational priority. These leadership behaviors may also strengthen trust and psychological safety by making it more acceptable for employees to express concerns, ask for assistance, and acknowledge uncertainty.

Second, healthcare organizations should develop structured opportunities that enable reflection, meaning reconstruction, and emotional processing. Healthcare professionals frequently encounter suffering, ethical challenges, adverse events, and emotionally demanding situations that may threaten their sense of purpose and professional identity [32,68]. Reflective practice groups, facilitated team discussions, clinical debriefings, and opportunities to discuss ethical concerns may help sustain experiences of meaningful work and professional connection, while also providing psychologically safer spaces in which difficult experiences can be discussed. Such initiatives should be integrated into routine organizational processes rather than implemented as isolated wellness activities.

Third, organizations should strengthen peer-support mechanisms and relational resources. Because workplace spirituality emphasizes community and belonging [17,18,19,20], supportive relationships among colleagues represent an important pathway through which healthcare professionals experience connection and protection during periods of stress. Peer-support programs may reduce isolation, facilitate adaptive coping, and provide opportunities for shared emotional processing following difficult clinical experiences. Their effectiveness is likely to depend on whether participation is voluntary, trusted, adequately supported, and embedded within a broader organizational culture that values openness and psychological health.

Fourth, healthcare organizations should promote cultures of learning rather than blame. Psychological safety requires that employees feel able to report concerns, discuss mistakes, and seek assistance without fear of punishment or humiliation [24]. Therefore, responses to errors and challenging situations should prioritize improvement, reflection, and collective learning rather than individual blame. Organizational responses to adverse events should distinguish learning-oriented accountability from punitive responses to human error. Such approaches may strengthen psychological safety and encourage speaking up, help-seeking, error reporting, and knowledge sharing, thereby supporting both workforce well-being and patient safety.

Finally, psychological safety should be incorporated into broader workforce sustainability strategies. Creating environments where healthcare professionals feel confident speaking up, questioning decisions, and acknowledging uncertainty is not only relevant for employee well-being but also essential for improving teamwork, learning, and the quality and safety of healthcare delivery in different cultural contexts [31]. From the perspective of the proposed framework, psychological safety represents a proximal mechanism through which broader organizational conditions may influence everyday employee behaviors and, ultimately, workforce outcomes. Consequently, interventions aimed at workforce sustainability should consider not only workload and staffing pressures but also whether organizational systems and leadership practices enable employees to use relational and psychological resources effectively.

## 6. Limitations and Future Research Directions

### 6.1. Limitations

Several limitations should be acknowledged. First, the proposed framework is conceptual and does not provide empirical evidence for the direction, magnitude, or causal nature of the relationships proposed. The sequential pathway from workplace spirituality through spiritual leadership, psychosocial safety climate, and psychological safety should therefore be interpreted as a theoretically informed proposition rather than an established causal process. In particular, workplace spirituality and spiritual leadership may be reciprocally related in practice, and future longitudinal research is required to determine temporal ordering.

Second, conceptual and measurement boundaries between the constructs require careful consideration. Workplace spirituality overlaps to some extent with related concepts such as meaningful work, organizational identification, organizational culture, belonging, ethical leadership, compassion, and work engagement. The present framework addresses this issue by retaining the established three-dimensional formulation of workplace spirituality and explicitly distinguishing it from these related constructs; however, empirical research will still need to establish discriminant validity using theoretically distinct and validated measures.

Third, the proposed multilevel relationships present methodological challenges. Workplace spirituality is primarily conceptualized as an employee-perceived experience, PSC as an organizational-level climate, psychological safety as a team-level or interpersonal perception, and well-being and burnout as individual-level outcomes. Cross-level relationships should therefore not be assumed without appropriate evidence of within-group agreement, aggregation, and temporal ordering. In particular, the proposed organizational-to-team translation from PSC to psychological safety requires empirical examination rather than being treated as automatic.

Finally, the framework may not operate uniformly across healthcare settings, professional groups, or cultural contexts. Organizational hierarchy, workload, leadership structures, professional norms, multidisciplinary interdependence, and cultural understandings of spirituality and communication may influence the strength and direction of the proposed relationships. The framework should therefore be regarded as a theoretically transferable model whose assumptions require empirical examination across diverse healthcare contexts rather than as a universal explanatory model.

### 6.2. Future Research Directions

Although research on workplace spirituality, psychological safety, psychosocial safety climate, and healthcare workforce well-being continues to expand, several important questions remain unresolved. Future research should therefore move beyond demonstrating associations between individual constructs and empirically examine the multilevel, temporal, and contextual processes proposed by the present framework. In particular, future studies should determine whether the proposed sequence from workplace spirituality to spiritual leadership and organizational practices, psychosocial safety climate, psychological safety, and workforce outcomes is supported by longitudinal and multilevel evidence, while also examining alternative and reciprocal explanations.

First, longitudinal research is needed to examine the temporal ordering of the proposed relationships. Existing research provides evidence of associations between workplace spirituality, leadership, psychosocial safety climate, psychological safety, and employee outcomes; however, cross-sectional designs cannot establish whether changes in one construct precede changes in another. Longitudinal studies should therefore examine whether changes in workplace spirituality and leadership practices are followed by changes in psychosocial safety climate and psychological safety and whether these changes are subsequently associated with changes in well-being, burnout, and other workforce outcomes. Such designs should also test competing temporal orderings, including whether spiritual leadership precedes changes in workplace spirituality, whether psychological safety subsequently influences perceptions of organizational climate, and whether employee well-being influences later perceptions of workplace spirituality, leadership, or psychological safety. This approach would allow researchers to distinguish the focal conceptual ordering from reciprocal or alternative temporal relationships.

Second, future research should employ multilevel designs that correspond to the conceptual levels of the framework. Because workplace spirituality, spiritual leadership, psychosocial safety climate, and psychological safety represent conceptually distinct phenomena that may operate at different levels, studies should avoid treating all variables as if they were individual-level constructs. Future research could examine workplace spirituality primarily as an employee-perceived experience, spiritual leadership at the leadership or organizational level, psychosocial safety climate at the organizational level, psychological safety at the team or interpersonal level, and well-being and burnout at the individual level. Multilevel structural equation modelling, cross-level modelling, and longitudinal multilevel approaches may provide appropriate methods for examining whether organizational-level conditions are associated with team-level psychological processes and, subsequently, individual-level outcomes. Where employee perceptions are aggregated to team or organizational levels, researchers should also establish appropriate within-group agreement and aggregation statistics.

Third, mixed-methods research is needed to examine how the proposed constructs are experienced and enacted in everyday healthcare practice. Quantitative research can test associations, temporal relationships, and multilevel pathways, whereas qualitative research can examine how healthcare professionals interpret concepts such as meaning, belonging, value alignment, compassion, trust, leadership, and psychological safety within specific clinical contexts. This is particularly important because workplace spirituality may not be experienced uniformly across professional groups or organizational settings. Qualitative and mixed-methods studies could therefore examine whether the three dimensions of workplace spirituality—meaningful work, sense of community, and alignment between individual and organizational values—are interpreted similarly across professions, clinical environments, and organizational contexts, and how these experiences interact with leadership and organizational practices.

Fourth, future research should examine contextual and cross-cultural boundary conditions. Experiences of spirituality, leadership, communication, psychological safety, and organizational values may vary across healthcare systems, professional groups, cultures, and organizational structures. Rather than assuming that the proposed relationships are universal, researchers should examine whether cultural values, professional norms, organizational hierarchy, workload, staffing conditions, and available organizational resources alter the strength or direction of specific relationships within the framework. Such research may clarify the circumstances under which value-based workplace experiences are successfully translated into organizational and interpersonal conditions and the circumstances in which these processes are disrupted.

Fifth, intervention and implementation research should examine whether the mechanisms identified in the framework can be influenced in practice. Future studies could evaluate leadership development programs, reflective practices, peer-support initiatives, team-based psychological safety interventions, and organizational strategies designed to strengthen meaningful work, belonging, value alignment, and psychologically healthy working conditions. Intervention studies should distinguish between interventions directed at individual experiences and those directed at leadership, organizational policies, or team processes, because improvements at one level may not necessarily produce corresponding changes at another level. Longitudinal follow-up would also be important for determining whether changes in psychological safety, psychosocial safety climate, and workforce well-being are sustained and whether intervention effects vary according to baseline working conditions and organizational context.

Finally, future empirical studies should directly compare the proposed framework with alternative theoretical models rather than testing the focal pathway in isolation. In particular, researchers could compare the proposed WPS → spiritual leadership → PSC → psychological safety → well-being sequence with models in which spiritual leadership precedes workplace spirituality, workplace spirituality and spiritual leadership have parallel relationships with PSC, or psychological safety contributes independently to well-being without requiring the full upstream sequence. Studies could also examine whether PSC and psychological safety operate as sequential, partially independent, or reciprocal processes and whether employee well-being subsequently influences perceptions of workplace spirituality, leadership, and organizational climate. Comparing these alternative specifications would help determine whether the proposed multilevel pathway provides explanatory value beyond direct, parallel, reverse-direction, or reciprocal models.

The framework therefore provides a set of empirically testable questions rather than an established causal or mediation model. Future studies should assess whether the proposed relationships represent direct associations, sequential indirect pathways, reciprocal processes, or context-dependent relationships. Longitudinal multilevel modelling and multilevel structural equation modelling may be particularly useful for examining these possibilities, provided that the temporal ordering, level of analysis, aggregation assumptions, and relevant contextual moderators are appropriately specified. Such research would provide a stronger basis for determining how value-based workplace experiences, leadership practices, organizational psychosocial conditions, and interpersonal psychological processes interact to influence healthcare professionals’ well-being and workforce sustainability.

## 7. Conclusions

Workplace spirituality may provide an important value-based foundation for understanding healthcare professionals’ well-being, particularly in work environments characterized by high emotional demands, professional hierarchy, clinical uncertainty, and workforce pressures. This conceptual paper proposes that the relationship between workplace spirituality and well-being is not necessarily direct, but may operate through organizational and interpersonal processes. Specifically, the framework positions spiritual leadership as an enabling mechanism through which value-based principles may be translated into organizational practices and psychosocial safety climate, which may in turn support psychological safety within healthcare teams.

By integrating workplace spirituality, spiritual leadership, psychosocial safety climate, and psychological safety, the framework provides a multilevel perspective on how meaning, community, and alignment between individual and organizational values may become connected to healthier workplace experiences. The contribution of the present paper should be understood as a conceptual integration rather than the development of a new standalone theory. The proposed relationships remain conceptual and require empirical testing, particularly through longitudinal and multilevel research that can examine directionality, reciprocal relationships, and contextual conditions. Nevertheless, the framework offers a basis for future research and for healthcare organizations seeking to foster workplaces characterized by meaning, trust, openness, psychological safety, and sustainable workforce well-being.

## Figures and Tables

**Table 1:** Conceptual Definitions and Levels of Analysis of Key Constructs.

Construct	Conceptual Definition	Primary Level of Analysis	Role in Proposed Framework	Distinction from Related Constructs
**Workplace spirituality**	Employees’ experience of meaningful work, a sense of community and connectedness, and alignment between personal and organizational values.	Primarily individual/perceptual, with organizational implications	Upstream value-based psychosocial condition	Focuses on meaning, belonging, connectedness, and values alignment rather than energy or task involvement.
**Spiritual leadership**	A leadership approach emphasizing vision, hope/faith, altruistic love, and shared purpose to foster meaning and belonging.	Leader/organizational	Enabling mechanism through which value-based principles may be translated into leadership behaviors and organizational practices	Represents leadership processes, rather than employees’ experience of workplace spirituality.
**Psychosocial safety climate (PSC)**	Shared perceptions that organizational policies, practices, leadership, and management priorities genuinely protect employees’ psychological health and address psychosocial risks.	Organizational/team climate	Organizational bridge between value-based conditions and interpersonal psychological safety	Concerns organizational priorities and systems for psychological health rather than meaning or interpersonal risk-taking.
**Psychological safety**	A shared belief that interpersonal risk-taking, including speaking up, asking questions, admitting mistakes, and seeking help, can occur without fear of negative interpersonal consequences.	Team/interpersonal	Proximal relational mechanism	Concerns the safety of interpersonal interactions rather than organizational commitment to psychological health.
**Work engagement**	A positive, fulfilling work-related psychological state characterized by vigor, dedication, and absorption.	Individual	Potential downstream outcome or related employee state	Concerns energy, dedication, and immersion in work, whereas workplace spirituality concerns meaning, community, and value alignment.
**Healthcare professionals’ well-being**	A broad state of positive psychological and occupational functioning reflecting healthy adaptation and functioning at work.	Individual	Distal positive outcome	Represents the principal employee-level outcome of the proposed pathway.
**Burnout/emotional exhaustion**	Occupational strain involving psychological depletion associated with prolonged exposure to demanding work conditions.	Individual	Negative well-being outcome	Represents occupational strain rather than a positive psychosocial resource or organizational condition.

**Table 2:** Suggested empirical operationalization and testing of the proposed framework.

Proposition	Proposed Relationship	Primary Level(s) of Analysis	Candidate Measurement Domain	Potential Empirical Test
P1	WPS as upstream value-based psychosocial condition	Individual; potentially aggregated	Meaningful work; community; value alignment	Longitudinal regression/multilevel modelling
P2	WPS → PSC through translation of values	Individual → organizational	WPS; PSC	Cross-level/longitudinal modelling
P3	SL as translation mechanism	Leadership/organizational	WPS; SL; PSC	Multilevel indirect-effect analysis/SEM
P4	PSC → psychological safety	Organizational → team	PSC; psychological safety	Cross-level modelling/SEM
P5	Psychological safety → well-being	Team → individual	Psychological safety; well-being/burnout	Longitudinal multilevel modelling
P6	Sequential indirect pathway	Cross-level	All framework constructs + moderators	Longitudinal multilevel modelling/SEM

Note: → = direction of hypothesized effect/relationship; WPS = Workplace Spirituality, PSC = Psychosocial Safety Climate, SL = Spiritual Leadership, SEM = Structural Equation Modelling.

## Data Availability

No new data were created or analyzed in this study. Data sharing is not applicable to this article.
